# The Impact of COVID-19 Pandemic on Quality of Life among General Population at Argolida regional unit

**DOI:** 10.1192/j.eurpsy.2022.957

**Published:** 2022-09-01

**Authors:** A. Gamvroula, S. Antonopoulos, E. Stratou, C. Toutouni, S. Aggelaina, G. Lampou, M. Saridi

**Affiliations:** General hospital Argolidas, General Hospital Argolidas, Argos, Greece

**Keywords:** Quality of Life, Covid-19 pandemic, Argolida

## Abstract

**Introduction:**

The impact of the Covid-19 on the quality of life (QOL) have been reported and highlighted by several research studies worldwide.

**Objectives:**

The primary objective of this study is to evaluate the impact of the COVID-19 pandemic on quality of life among Greek general population of Argolida, taking into consideration the socio-demographic characteristics.

**Methods:**

Information on the socio-demographic characteristics and Covid related data of the respondents was collected by a questionnaire including age, gender, education level, marital status, health status, smoking history, sedentary lifestyle, job status. A 5-point Likert scale (MVQOLI) was used to examine the QOL. Comparisons on the variables were performed using Kruskal-Wallis H Test and x2 test, using SPSS Statistics (version 20).

**Results:**

A total of 620 Greek adults (Females n =381) were requested to answer by filling the questionnaire or Google Form. The results showed statistically significant differences in higher level QOL depending on a number of variables that are presented in the table. Table Demographic characteristics of the study with higher level QOL

**Table:** 

Sample Characteristics	p Value
Gender : Female	0,003
Marital status: Married /cohabitation	0,011
Level of education : High	0,000
Health status: Non psychiatric disease	0,022
Smoking status: Never smoked & ex-smoker	0,043
Sedentary life style	0,002
Non covid-19 affected	0,001
Confidence in the health system	0,001
Confidence in health workers	0,001

**Conclusions:**

The results showed that female sex, married, higher educated respondents, non smokers were significantly associated with higher QOL. The findings can certainly offer guidelines in developing programs and interventions for all domains of QOL.

**Disclosure:**

No significant relationships.

